# Prediction of Host-Derived miRNAs with the Potential to Target *PVY* in Potato Plants

**DOI:** 10.3389/fgene.2016.00159

**Published:** 2016-09-14

**Authors:** Muhammad S. Iqbal, Muhammad N. Hafeez, Javed I. Wattoo, Arfan Ali, Muhammad N. Sharif, Bushra Rashid, Bushra Tabassum, Idrees A. Nasir

**Affiliations:** ^1^Centre of Excellence in Molecular Biology, University of the PunjabLahore, Pakistan; ^2^Faculty of Life Sciences, University of Central PunjabLahore, Pakistan; ^3^Institute of Molecular Biology and Biotechnology, The University of LahoreLahore, Pakistan

**Keywords:** potato virus Y, microRNA, miRanda, RNA-22, RNA hybrid, target prediction, *Solanum tuberosum*, potato

## Abstract

*Potato virus Y* has emerged as a threatening problem in all potato growing areas around the globe. *PVY* reduces the yield and quality of potato cultivars. During the last 30 years, significant genetic changes in *PVY* strains have been observed with an increased incidence associated with crop damage. In the current study, computational approaches were applied to predict Potato derived miRNA targets in the *PVY* genome. The *PVY* genome is approximately 9 thousand nucleotides, which transcribes the following 6 genes:*CI, NIa, NIb-Pro, HC-Pro, CP, and VPg*. A total of 343 mature miRNAs were retrieved from the miRBase database and were examined for their target sequences in *PVY* genes using the minimum free energy (mfe), minimum folding energy, sequence complementarity and mRNA-miRNA hybridization approaches. The identified potato miRNAs against viral mRNA targets have antiviral activities, leading to translational inhibition by mRNA cleavage and/or mRNA blockage. We found 86 miRNAs targeting the *PVY* genome at 151 different sites. Moreover, only 36 miRNAs potentially targeted the *PVY* genome at 101 loci. The *CI* gene of the *PVY* genome was targeted by 32 miRNAs followed by the complementarity of 26, 19, 18, 16, and 13 miRNAs. Most importantly, we found 5 miRNAs (miR160a-5p, miR7997b, miR166c-3p, miR399h, and miR5303d) that could target the *CI, NIa, NIb-Pro, HC-Pro, CP*, and *VPg* genes of *PVY*. The predicted miRNAs can be used for the development of *PVY*-resistant potato crops in the future.

## Introduction

*PVY* has emerged as one of the most alarming pathogens in potatoes around the world; it affects the yield and quality of potatoes by inducing ringspot disease (Lorenzen et al., 2006; Ali et al., 2016). In plants, *PVY* can be transmitted via vegetative propagation, seed tubers and aphids (Robert et al., 2000). In recent years, biotechnology has opened new horizons to combat *PVY* by introducing transgenic varieties of *Solanum tuberosum* (McCue et al., 2012). The infection cycle of a potyvirus begins when the viral particle enters the cell via a wound or during feeding by its vector aphid (Filipowicz and Hohn, 1996; Bailey-Serres, 1999).

**Figure 1:**
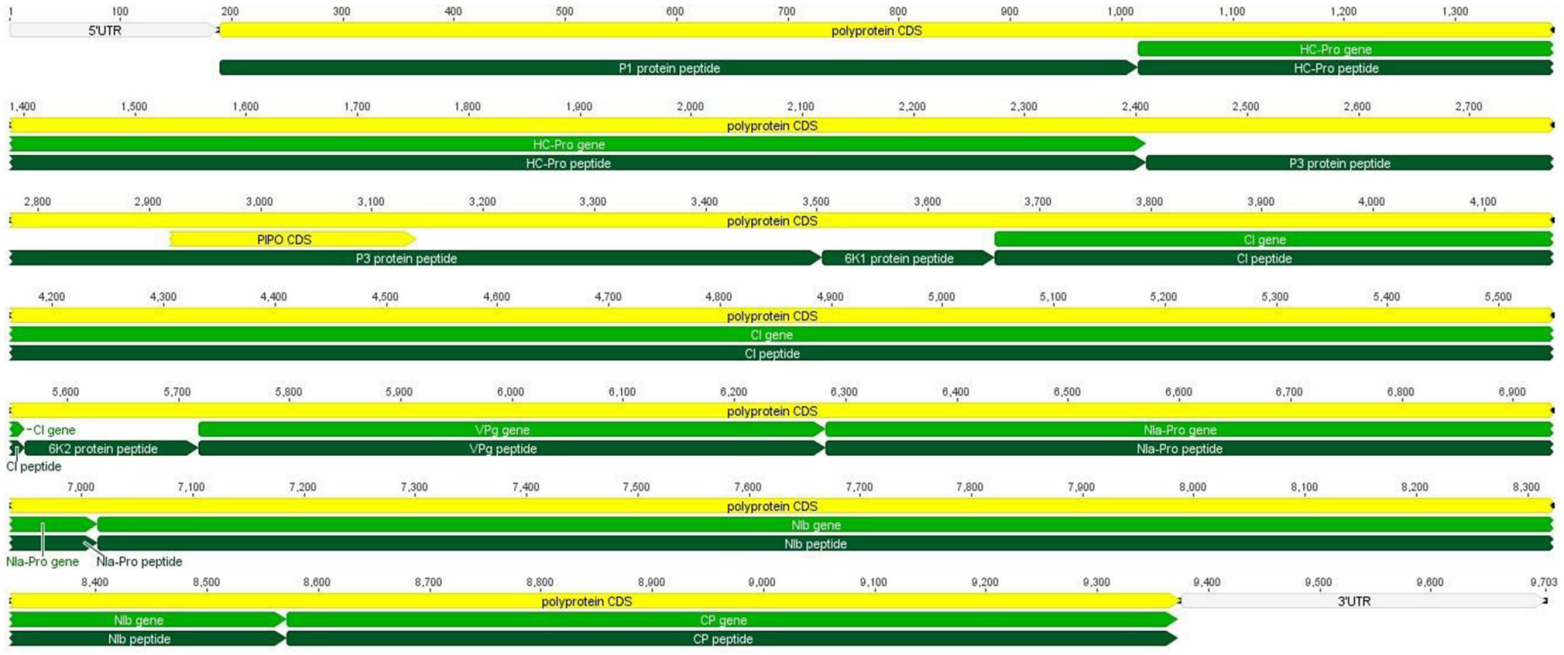
**Genome organization of *PVY***. Six genes (*CI, NIb, HC-Pro, NIa-Pro, VPg, CP*) of *PVY* are shown along with their nucleotide length. The whole genome is translated as a single polypeptide. Yellow color indicates polypeptides, light green indicates specific genes and dark green represents the corresponding protein.

*PVY* is a positive sense, single-stranded RNA virus, belong to the *Potyviridae* family and genus potyvirus. Its genome size is 9.7 kb, and it has a polyadenylated tail at the 3′ terminus and the VPg gene at the 5′ terminus. The virus releases its RNA into the cell cytoplasm, and this ssRNA uses the host ribosomal machinery for translation (Teycheney et al., 2000). Transcription factor elF4E is present on the 5′ end of the viral genome and helps in translations it does in most eukaryotes (Ruffel et al., 2002). *PVY* expresses its genome as a single large polypeptide that cleaves into 3 virus-specific proteases (Figure 1, Glais et al., 2002). In addition to CP, HC-Pro, and VPg were also found to be covalently attached to the *PVY* RNA genome (Karasev and Gray, 2013). The genome is encapsulated by nearly 2000 copies of CP (Coat Protein). The complete genome is transcribed by a single Open Reading Frame (ORF), which encodes a large polyprotein of approximately 3000–3500 amino acids ultimately cleaved by 3 viral encoded proteins (P1, Hec-Pro, and NIa-Pro). Ten mature proteins are produced after cleavage (Verchot et al., 1991).

*Solanum tuberosum* has an active immunity in the form of miRNAs that fights against the *PVY* infection. This immunity is rendered by 21–24 nt small regulatory RNAs, i.e., microRNAs (miRNAs) (Brigneti et al., 1998). miRNAs are short endogenous single-stranded RNA molecules (21–24 nt) that regulate gene expression patterns in plants and animals (Rogers and Chen, 2013) and are derived from stem loop regions of nuclear transcripts or transcripts of endogenous plant loci (Pillai et al., 2007). miRNAs are usually synthesized in the form of imperfect hairpin structures (Kim, 2005). The pri-miRNA transcript is cleaved to pre-miRNA by the Dicer-like 1 (DCL1) protein of RNase type III (Mlotshwa et al., 2008) and produces a distinctive 21-nucleotide, double-stranded RNA. This duplex is further exported into the cytoplasm by HASTY and is methylated at the 3′ end by HEN1 (Jones-Rhoades et al., 2006). In the cytoplasm, miRNAs direct endogenous plant transcripts for translational repression or mRNA degradation in a sequence-specific manner (Valencia-Sanchez et al., 2006). The significant role of small RNAs in plants' defense against viruses has been reported in many studies (Witkos et al., 2011).

The goal of this study is to predict the most effective miRNAs that induce resistance against *PVY*. In this article, a non-conventional miRNA-based approach was designed to assess viral resistance in potato plants. We used the most efficient bioinformatic tools for the identification of potential miRNA target sites within the *PVY* genome. The artificially designed miRNAs can be used to transform *Solanum tuberosum* to combat *PVY* infection.

## Methodology

### Data source

Mature microRNA sequences of potato were obtained from the miRNA database http://www.mirbase.org/cgi-bin/browse.pl. The target genome sequence was retrieved from the NCBI nucleotide database (http://ncbi.nlm.nih.gov) bearing accession number AB714135.

### Sequence analysis

Genome organization, ORFs and nucleotide distribution of *PVY* genome were calculated through CLC Genomics Workbench v8.

### Target prediction

*In silico* tools often predict numerous target sites within the target sequence, and only a limited number of them have been validated experimentally. In plants, miRNA-mRNA attachment is less complicated than it is in animals and it mostly depends on a higher quality match between the target sequence and the miRNA than it does in animals (Witkos et al., 2011). Researchers have analyzed algorithms for their accuracy and efficiency, and some have reported their results as a guideline to be used as a stepping stone (Witkos et al., 2011). Furthermore, three different software programs were selected for the miRNA target prediction on the basis of their reported performance in the most recent literature. miRanda and RNAhybrid were among the best tools recommended for target prediction, while our third selection, RNA22, utilized a different set of analytic approaches (pattern recognition) to fully analyze the miRNA attachment with the target sequence and reduce false-positive results to some extent. Potential miRNAs targets detected are the intersection set of the results from three softwares.

### miRanda

As the first miRNA target prediction software, miRanda (John et al., 2004) is the most frequently used algorithm software today for both plants and animals (John et al., 2004; Liu et al., 2015). As its script is quite basic in function for finding attachment sites, there is the possibility of producing false positives for attachment sites in the target genome. This tool selects its target match using the following three properties:(1) complimentary sequence;(2) free energy of RNA-RNA duplex and target conservation in related genomes; and (3) accounting for the final result, which is a weighted sum of the match and mismatch scores for base pairs and gap penalties. The miRanda Algorithm was downloaded from the source website (http://cbio.mskcc.org/miRNA2003/miranda.html), and the *PVY* genome was assessed to determine whether there were any possible targets for *Solanum tuberosum* miRNAs. The analysis was performed by miRanda at its default settings (−*E* = −20 kcal/mol, Score threshold = 50, energy threshold = −20 kcal/mol, scaling parameter to *z* = 2, gap-open penalty to *X* = −2, gap-open penalty to *z* = −8).

### RNAhybrid

RNAhybrid predicts miRNA and mRNA hybridization based on minimum free energy and site complementary. “Tapirhybrid,” another tool rated as one of the best tools for miRNA target prediction (Srivastava et al., 2014), uses the same algorithm as RNAhybrid (as mentioned on the Tapirhybrid official webpage). RNAhybrid has also been used to locate an exact match for a miRNA target in plants (Hariharan et al., 2005). miRNAs against a *PVY* genome attachment were analyzed by RNAhybrid (http://bibiserv.techfak.uni-bielefeld.de/rnahybrid) (Krüger and Rehmsmeier, 2006) at an energy threshold of −20 kcl/mol, and other filters were set to the default parameters. The software reported some results of mfe that deviated from the threshold values, but these results were excluded from the final list (Table, please see Supplementary File). We used this software to eliminate any possible false positive attachments shown by miRanda. The *E*-value was set to −20 kcal/mol, and the remainder of the parameters were set to default (for more details, please see Table in Supplementary File).

### RNA22 v2.0 interactive predictions (https://cm.jefferson.edu/rna22v2/)

The Rna22 algorithm (Miranda et al., 2006) predicts target patterns that are statistically significant miRNA motifs created after a sequence analysis of known mature miRNAs. RNA22 searches for reverse complement sites of patterns within mRNAs of interest and determines sites with many aligned patterns (so-called “hot spots”). The next step is the identification of miRNAs that are likely to bind to these sites. This approach also enables the identification of sites targeted by yet-undiscovered miRNAs. The minimum number of base-pairs between miRNA and mRNA, the maximum number of unpaired bases and the free energy cutoff were the user-defined parameters.

### Phylogenetic analysis

The complete sequence of the corresponding *PVY* polyprotein was retrieved from NCBI, and homologous sequences were obtained using the BLAST tool with the DELTA-BLAST (Domain Enhanced Lookup Time Accelerated Blast) algorithm referenced in protein databases. Fifty of the most homologous sequences were retrieved and aligned using the Muscle alignment tool (Edgar, 2004) along with the query sequence. Aligned sequences were used to build a phylogenetic tree using a poison algorithm by NCBI Genome Workbench.

## Results

Different families of miRNAs of *Solanum tuberosum* have considerable potential to target potato Virus-Y. miRNA families, such as miR166c-3p, miR482e-5p, miR5303a, miR5303d, miR8004, miR8032b-5p, miR8032c, miR8032e-5p, miR162b-3p, miR164-3p, miR160a-5p, miR8011a-5p, miR8018, and miR482e-5p, were found to have more potential to target *PVY* at multiple loci. The *CI* gene was targeted by 32 different miRNAs, followed by *Nib, HC-Pro, NIa-Pro, VPg*, and *CP*, which were targeted by 26, 19, 18, 16, and 13 miRNAs, respectively (Figure 1).

### miRNAs targeting CIP (cylindrical inclusion protein) gene

**Figure 2:**
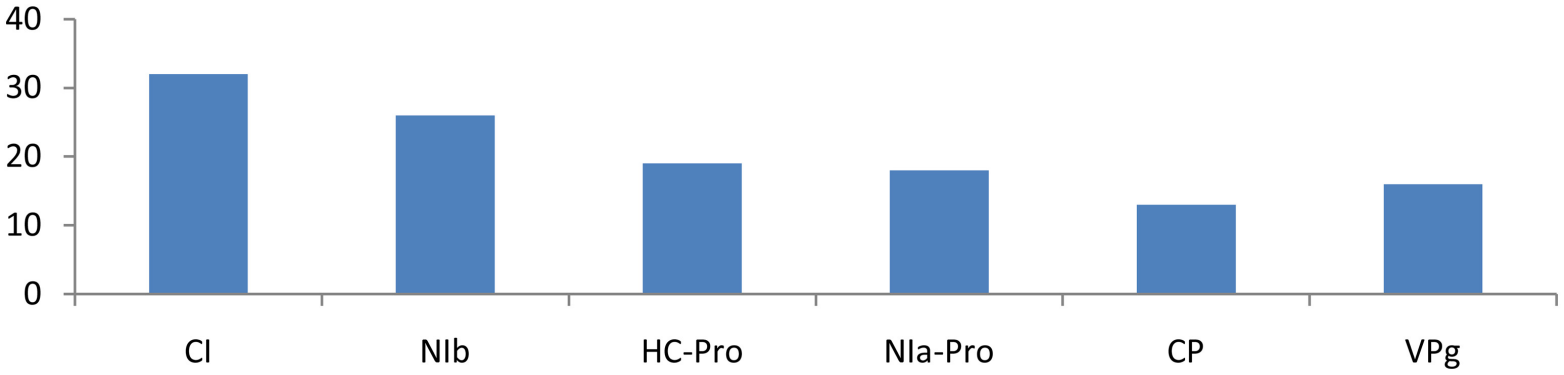
**Number of putative miRNA targets of *CI, NIb, HC-Pro, NIa-Pro, CP*, and *VPg* of Potato Virus Y (*PVY*)**. Bars show number of hits of putative miRNAs targets above threshold values calculated by various software (miRanda, RNA22, and RNAhybrid).

The CIP gene showed an interaction with 32 miRNAs (miR5303a, miR5303d, miR8032b-5p, miR8032c, miR8032e-5p, miR166c-3p, miR482e-5p, miR8004, miR156d-3p, miR160a-3p, miR167b-5p, miR319a-3p, miR393-3p, miR399h, miR482d-5p, miR7980a, miR8032b-3p, miR8032d-3p, miR160a-5p, miR162b-3p, miR164-3p, miR166d-5p, miR167b-3p, miR167d-3p, miR397-5p, miR399l-5p, miR408a-3p, miR7996c, miR7997b, miR8018, and miR8032f-3p); Eight of these miRNAs(miR166c-3p, miR482e-5p, miR5303a, miR5303d, miR8004, miR8032b-5p, miR8032c, and miR8032e-5p) were shown to have multiple loci interactions at different nucleotide positions (Figure 2). Of note, miR166c-3p and miR482e-5p targeted the CIP gene at 7 positions, followed by miR5303a, miR5303d and miR8004, which targeted the gene at 5 positions, while miR8032b-5p, miR8032c, and miR8032e-5p targeted the gene at 3 different loci.

### miRNAs targeting NIb (nuclear inclusion protein b) gene

**Figure 3:**
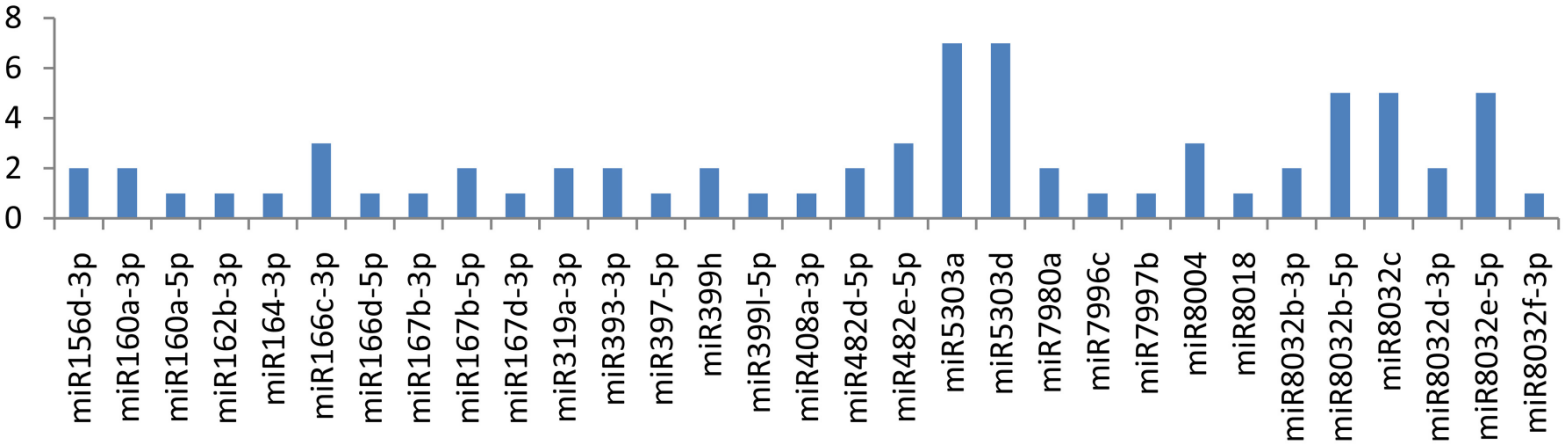
**miRNA families and the number of putative targets to the CI gene of the*Potato Virus Y (PVY*)**. Bars show the number of hits for individual miRNAs of *Solanum tuberosum* to *PVY* genome.

The *NIb* gene was targeted by 20 different miRNAs (miR156d-3p, miR160a-5p, miR162b-3p, miR164-3p, miR166c-3p, miR166d-5p, miR167b-3p, miR167d-3p, miR169b-3p, miR169c-3p, miR5303a, miR5303d, miR7991a, miR7992-3p, miR7992-5p, miR7997b, miR8000, miR8006-5p, miR8011a-5p, and miR8018). It is imperative to discuss that only one miRNA (miR162b-3p) targeted the NIb gene at four different positions, while the other three miRNAs(miR164-3p, miR7992-3p, and miR8000) targeted the gene at two loci (Figure 3). The remaining16 of the 20 miRNAs that specifically targeted the Nib gene of *PVY* targeted the gene at a single locus.

### miRNAs targeting *HC-Pro* (helper component proteinase) gene

**Figure 4:**
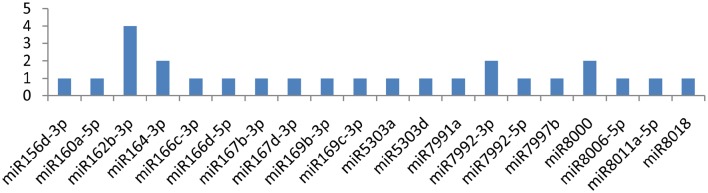
**miRNA families and the number of putative targets to the *NIb* gene of Potato Virus Y (*PVY*)**. Bars show the number of hits for individual miRNAs of *Solanum tuberosum* to *PVY* genome.

**Figure 5:**
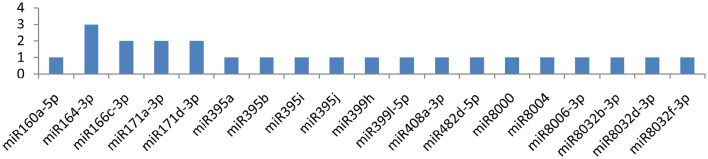
**miRNA families and the number of putative targets to the HC-Pro gene of Potato Virus Y (*PVY*)**. Bars show the number of hits for individual miRNA of *Solanum tuberosum* to *PVY* genome.

Similarly, miRNAs, such as miR164-3p, miR166c-3p, miR171a-3p, and miR171d-3p, targeted the *HC-pro* gene at multiple loci, while the gene was attacked by 19 miRNAs (**Figure 5**). miR164-3p targeted the HC-Pro gene at 3 different loci, and each miRNA(miR166c-3p, miR171a-3p, miR171d-3p) targeted HC-Pro at 2 different positions (Figure 4). Other than these four miRNAs, miR160a-5p, miR395a, miR395b, miR395i, miR395j, miR399h, miR399l-5p, miR408a-3p, miR482d-5p, miR8000, miR8004, miR8006-3p, miR8032b-3p, miR8032d-3p, and miR8032f-3p targeted the *HC-Pro* gene at a single locus.

### miRNAs targeting the NIa-Pro (C-terminal proteinase domain of NIa) gene

**Figure 6:**
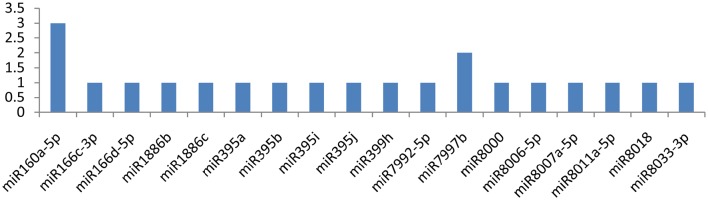
**miRNA families and the number of putative targets to the *NIa-Pro* gene of *Potato Virus Y (PVY*)**. Bars show the number of hits for individual miRNA of *Solanum tuberosum* to *PVY* genome.

The *NIa-pro* gene was potentially targeted by miR160a-5p and miR7997b at more than one locus (**Figure 6**). The former miRNA targeted the NIa-Pro gene at three loci, and the latter targeted at two loci. The *NIa-Pro* gene was observed to be attacked by 18 different miRNAs; only three of the above-mentioned miRNAs targeted the gene at multiple loci, while the other 15 miRNAs (miR166c-3p, miR166d-5p, miR1886b, miR1886c, miR395a, miR395b, miR395i, miR395j, miR399h, miR7992-5p, miR8000, miR8006-5p, miR8007a-5p, miR8011a-5p, miR8018, and miR8033-3p) targeted at a single locus as shown in Figure 5.

### miRNAs targeting(coat protein) and Vpg (viral genome-linked protein) genes

**Figure 7:**
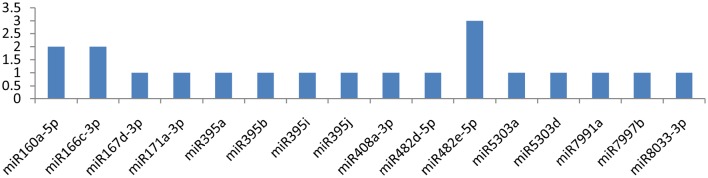
**miRNA families and the number of putative targets to the VPg gene of *Potato Virus Y (PVY*)**. Bars show the number of hits for individual miRNAs of *Solanum tuberosum* to *PVY* genome.

**Figure 8:**
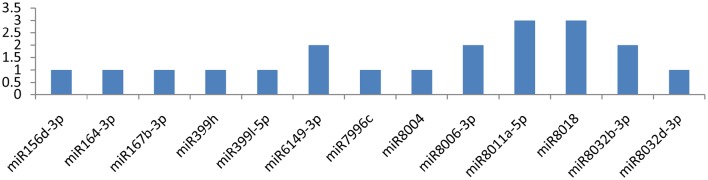
**miRNA families and the number of putative targets to the CP gene of *Potato Virus Y (PVY*)**. Bars show the number of hits for individual miRNAs of *Solanum tuberosum* to *PVY* genome.

Among the 29 miRNAs targeting CP and VPg genes, only seven miRNAs targeted them at multiple loci. miR8011a-5p and miR8018 targeted the CP gene at six different loci (Figure 8). The other three miRNAs (miR6149-3p, miR8006-3p, and miR8032b-3p) targeted the CP gene at two different positions (Figure 6), and remaining eight miRNAs (miR156d-3p, miR164-3p, miR167b-3p, miR399h, miR399l-5p, miR7996c, miR8004, and miR8032d-3p) attached at a single position. While the VPg gene was targeted by miR482e-5p at three loci, the other two miRNAs (miR160a-5p and miR6149-3p) targeted the VPg gene at two different loci (Figure 7). It is also important to discuss the miRNAs (miR167d-3p, miR171a-3p, miR395a, miR395b, miR395i, miR395j, miR408a-3p, miR482d-5p, miR5303a, miR5303d, miR7991a, miR7997b, and miR8033-3p) that potentially targeted the *VPg* gene at a single locus (Figure 7). It is also important to mention that five miRNAs (miR160a-5p, miR7997b, miR166c-3p, miR399h, and miR5303d) could target the *CI, NIb, HC-Pro, NIa-Pro, CP*, and *VPg* genes of *PVY* at multiple loci.

### Most effective miRNAs against potato virus Y (PVY)

**Figure 9:**
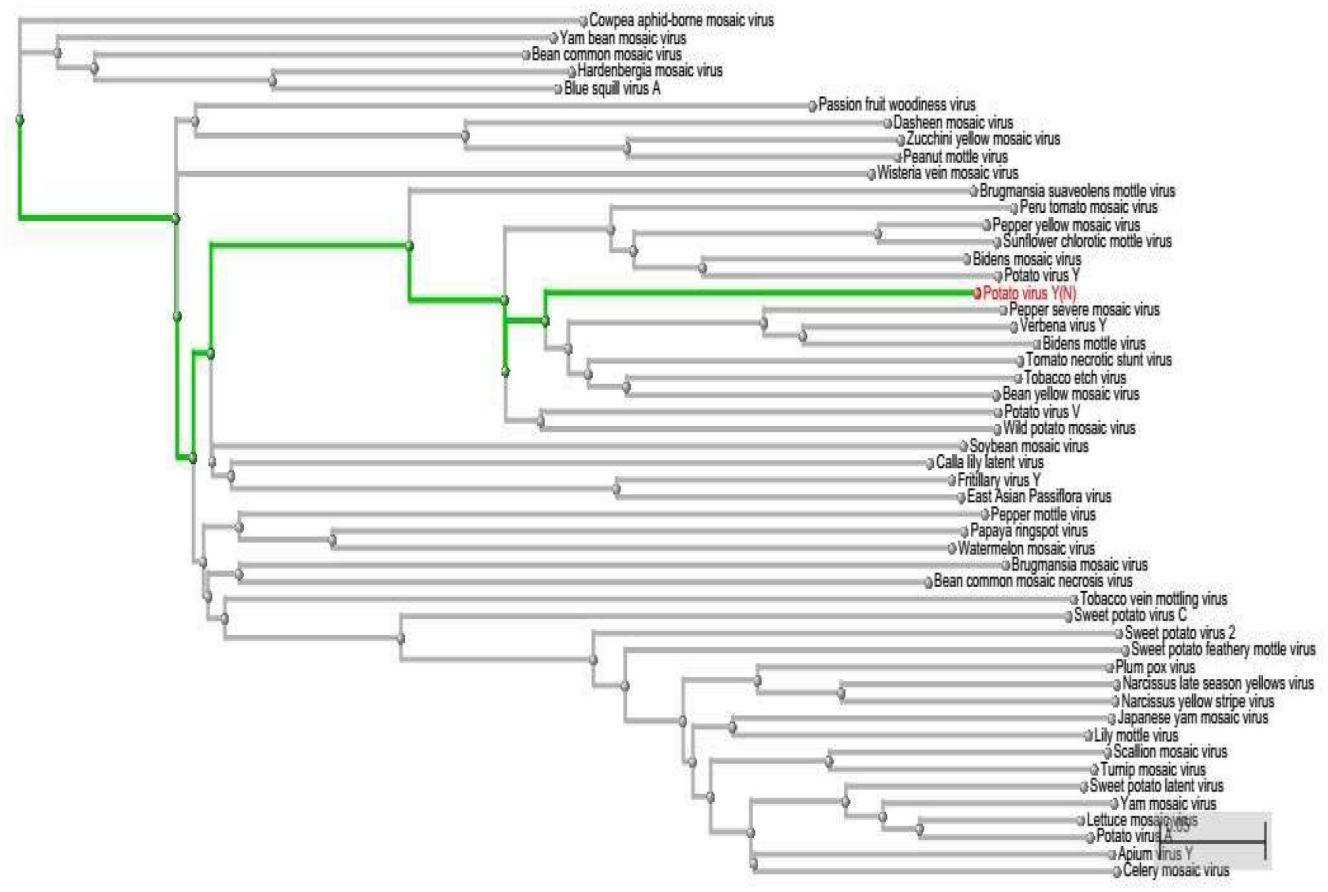
**Phylogenetic Tree represented in a rectangular cladogram shows the reference sequence of *PVY* (highlighted in green) and other closely related and distant virus species**.

Using this computational approach of miRNA-mRNA nucleotide match and mismatch, we have assessed the possible off-targets of miRNAs by phylogenetically analyzing the *PVY* sequence. On the one hand, we have short-listed 5 miRNAs (miR160a-5p, miR7997b, miR166c-3p, miR399h, and miR5303d) that could target the genes of *PVY* at multiple loci. On the other hand, on the basis of the polyprotein delta blast, we found other virus stains, which were genetically similar to *PVY* (Figure 9). This approach is helpful for planning effective experimental designs against specific or non-specific virus species.

## Discussions

The current study used bioinformatic tools to identify the miRNAs of *Solanum tuberosum*, which targeted *PVY*^N^. The computational analysis concluded that 86 miRNAs targeted the *PVY* genome at 151 different positions, and only 36 of the 86 miRNAs targeted the CI, NIb, HC-Pro, NIa-Pro, CP, and VPg genes of *PVY* at 101 loci.

### Cylindrical inclusion protein

The CI (cylindrical inclusion protein) gene was targeted by 32 miRNAs. The CI gene consists of 1901 nucleotides encoding cylindrical inclusion protein. The gene has helicase activity (Lain et al., 1990; Eagles et al., 1994) and facilitates cell-to-cell movement (Carrington et al., 1998). This study found 7 miRNAs, which potentially targeted the 1901-long nucleotide CI gene at multiple loci. miRNAs interfere with the replication and translation of the CI gene. A total of 32 miRNAs targeted the *CI* gene at 58 different positions and rendered CI functionless. The critical analysis also indicated that two miRNAs, miRNA5303a, and miRNA5303b, has great potential to target the CI gene at 14 different positions. miRNAs targeting the *CI* gene cannot function properly, and hence, the genome of *PVY* remains harmless to the potato plant.

### Nuclear inclusion protein b

The *NIb* (nuclear inclusion protein b) gene was targeted by 26 miRNAs, and four of the miRNAs (miR162b-3p, miR164-3p, miR7992-3p, and miR8000) were found to be more important because they targeted the gene at 10 different positions. miR162b-3p potentially targeted the gene at four multiple positions, and the other three miRNAs (miR164-3p, miR7992-3p, and miR8000) targeted the gene at 6 possible sites. NIb gene nucleotides are present at the 5′ terminus from 7015 to 8571 nt. The gene encodes the RNA-dependent RNA polymerase, and it is involved in viral replication (Hong and Hunt, 1996). NIb also interacts with NIa-Pro protein, meaning that they influence each other's function or their products work in close proximity (Li et al., 1997). In addition, the interaction of NIa-pro with NIb provides a clue that NIa-pro is also involved in viral replication, probably recruiting NIb to the site of replication through a protein-protein interaction (Fellers et al., 1998). Using bioinformatic tools, we found 26 miRNAs targeting the NIa gene at 38 multiple loci, which may considerably disturb its function.

### Helper component proteinase

In this study, we found 19 miRNAs (miR164-3p, miR166c-3p, miR171a-3p, miR171d-3p, miR160a-5p, miR395a, miR395b, miR395i, miR395j, miR399h, miR399l-5p, miR408a-3p, miR482d-5p, miR8000, miR8004, miR8006-3p, miR8032b-3p, miR8032d-3p, and miR8032f-3p) that potentially targeted the *HC-Pro* (helper component proteinase) gene. Among the 19 miRNAs, 4 (miR164-3p, miR166c-3p, miR171a-3p, and miR171d-3p) were found to be more critical because of their potential interaction at nine loci of the *HC-Pro* gene. The product of *HC-Pro* is proteinase (Carrington et al., 1989a,b), and similarly to Nib, it also facilitates cell-to-cell transmission (Klein et al., 1994; Rojas et al., 1997), genome replication (Atreya et al., 1992; Restrepo-Hartwig and Carrington, 1994) and Aphid transmission (Pirone and Thornbury, 1984). Of note, HC-Pro is found to be a suppressor of RNA silencing (Klein et al., 1994; Kasschau and Carrington, 1998; Rovere et al., 2002). It mediates its own cleavage from polyprotein and may be considered the main gene of the viral genome, which helps the virus to escape from the plant's immune response (Dougherty and Carrington, 1988). We found 19 miRNAs, which targeted *HC-Pro* at 24 different positions, making the plant naturally resistance against *PVY* infection.

### C-terminal proteinase domain of NIa

The current bioinformatic approach revealed 18 potential miRNAs that targeted the NIa-pro (C-terminal proteinase domain of NIa) gene at multiple loci. Their potential attachment to NIa-Pro not only interferes with the normal functioning of NIa-Pro but is also responsible for the abnormal activity of Nib because both genes work in collaborative manner. We found that only two miRNAs (miR160a-5p and miR7997b) interact with NIa-pro at five different positions. The other 16 miRNAs targeted this gene at a single locus. Proteinase by nature cleaves approximately two-thirds of the viral polypeptide at multiple positions to make them functional (Riechmann et al., 1992). There are two other proteinases (P1 and HC-Pro), which play a critical role in making the viral protein functional (Shukla and Ward, 1989). A total of 18 miRNAs were found that have the potential to silence NIa proteinase, which can render it functionless.

### Coat protein

The *CP* (coat protein) gene was targeted by 13 miRNAs. The CP encodes the capsid protein, which encapsulates the single-stranded RNA genome of *PVY*. It also facilitates cell-to-cell and long-distance movement (Dolja et al., 1995; Mahajan et al., 1996), genome amplification (Missiou et al., 2004), and Aphid transmission (Brigneti et al., 1998). Several attempts have been made to develop transgenic plants with the CP gene (Merits et al., 1998; Dan et al., 2009; Zhu et al., 2009; Tabassum et al., 2011). Using the bioinformatic approach, we found 5 miRNAs, which potentially targeted the 800-long nucleotide CP gene at multiple loci. miRNAs interfere with the replication and translation of the CP gene and ultimately render the genome of *PVY* functionless. A total of 13 miRNAs targeted the CP gene at 20 different positions. Two microRNAs, miR8011a-5p and miR8018, were also able to target the CP gene at six different positions.

### Viral genome-linked protein

We found 16 miRNAs with potential to inactivate the *Vpg* (viral genome-linked protein) gene.

The current study revealed that miR482e-5p, miR160a-5p, and miR166c-3p cleaved the Vpg gene at 7 positions, while 16 microRNAs targeted this gene at 20 different loci. The *VPg* gene encodes the viral genome-linked protein, which facilitates RNA replication (Schaad et al., 1996; Rajamäki and Valkonen, 1999), cell-to-cell movement (Rajamäki and Valkonen, 1999; Léonard et al., 2000), and forms a complex with eukaryotic translation initiation factor, eIF(iso)4E (Wittmann et al., 1997; Baulcombe, 2004). We found 16 miRNAs that have the potential to interrupt the normal functioning of this gene.

Putative miRNAs targets were selected on the basis of the miRanda, RNA22, and RNAhybrid algorithm (Re). Apart from perfect complementarities in miRNAs and their targeted regions with no mismatch, up to 1–24 nucleotides were focused upon, and only un-gapped miRNA-mRNA predicted targeted models were considered as best hits. Finally, we selected only those miRNAs that were found to be potential targets by all three software.

Controlling for viral infection, following viral mRNA degradation is the simplest approach. Niu and colleagues used a 273 bp sequence of Arabidopsis miR159a per-miRNA transcript expression amiRNAs against the viral suppressor genes P69 and HC-Pro to generate resistance against *Turnip yellow mosaic virus* and *Turnip mosaic virus* infection, respectively. Here, we may use miRNAs against *PVY* in transgenic plants.

## Conclusions

This study provides a better way to computationally analyze the best-candidate miRNAs against viruses, prior to cloning. As our approach allows a narrow-range of match-mismatch in microRNA-mRNA attachment, it screens most of the falsely predicted attachments. The phylogenetic tree helps to determine whether there is any possibility for cloned miRNAs to act off-target and affect other virus species. The more viral species that are discovered to be closer to *PVY*, are more likely to hit by cloned miRNA.

## Author contributions

The main idea was developed by MI and bioinformatics analysis were done by MI, MH, and AA, data interpretation was done by MS and JW and manuscript was written and proof-read jointly by all authors.

### Conflict of interest statement

The authors declare that the research was conducted in the absence of any commercial or financial relationships that could be construed as a potential conflict of interest.
